# Physician experiences of electronic health record interoperability and its practical impact on care delivery in the English NHS: a cross-sectional survey study

**DOI:** 10.1136/bmjopen-2024-096669

**Published:** 2025-06-10

**Authors:** Edmond Li, Olivia Lounsbury, Mujtaba Hasnain, Hutan Ashrafian, Ara Darzi, Ana Luisa Neves, Jonathan Clarke

**Affiliations:** 1 Global Digital Health Unit, Department of Primary Care and Public Health, Imperial College London, London, UK; 2 Institute of Global Health Innovation, Department of Surgery and Cancer, Imperial College London, London, UK; 3 School of Medical Sciences, University of Manchester, Manchester, UK; 4 Center for Health Technology and Services Research, Department of Community Medicine, University of Porto, Porto, Portugal; 5 Centre for Mathematics of Precision Healthcare, Department of Mathematics, Imperial College London, London, UK

**Keywords:** Electronic Health Records, Health & safety, Health informatics, Health Services, Digital Technology

## Abstract

**Abstract:**

**Background:**

The lack of interoperability has been a well-recognised limitation associated with the use of electronic health records (EHR). However, less is known about how it manifests for frontline NHS staff when delivering care, how it impacts patient care and what its implications are on care efficiency.

**Objectives:**

(1) To capture the perceptions of NHS physicians regarding the current state of EHR interoperability, (2) to investigate how poor interoperability affects patient care and safety and (3) to explore the effects it has had on care efficiency in the NHS.

**Methods:**

An online Qualtrics survey was conducted between June and October 2021 to explore how NHS physicians perceived the present state of interoperability among EHR in service, its effects on patient safety and its impact on care efficiency in NHS healthcare facilities. Recruitment was performed via convenience sampling and snowballing in collaboration with contacts at Health Education England deaneries and the Royal College of General Practitioners. Descriptive statistics were used to report any notable findings observed.

**Results:**

A total of 636 NHS physicians participated, of which 218 (34.3%) completed the survey fully. Participants reported that EHR interoperability is rudimentary across much of the NHS, with limited ability to read but not edit data from within their organisation. Negative perceptions were most pronounced among specialties in secondary care settings and those with less than 1 year of EHR experience or lower self-reported EHR skills. Limited interoperability prolonged hospital stays, lengthened consultation times and frequently necessitated repeat investigations to be performed. Limited EHR interoperability impaired physician access to clinical data, hampered communication between providers and was perceived to threaten patient safety.

**Conclusion:**

As healthcare data continues to increase in complexity and volume, EHR interoperability must evolve to accommodate these growing changes and ensure the continued delivery of safe care. The experiences of physicians provide valuable insight into the practical challenges limited interoperability poses and can contribute to future policy solutions to better integrate EHR in the clinical environment.

**STRENGTHS AND LIMITATIONS OF THIS STUDY**

- This is the first study attempting to quantify the frequency and nature of electronic health record (EHR) interoperability challenges routinely encountered by NHS doctors, map where these issues typically arise along clinical pathways and examine the impact it has on care delivery and productivity.
- Study responses were derived from a relatively large sample size of NHS doctors surveyed across England, including those working in hospital and community-based clinical settings, differing specialties and possessing a range of clinical training experience.
- Convenience sampling and snowballing recruitment approaches used may have resulted in some self-selection bias for study participants who may be more prone to sharing more polarising experiences concerning the study topic and thus not be representative of most NHS doctors.
- Higher survey attrition rates among certain subgroups of participants may have resulted in an underrepresentation of the EHR interoperability challenges they routinely encounter.
- This study being performed during the COVID-19 pandemic may have captured views regarding interoperability issues which may not necessarily be representative of routine EHR usage experienced by NHS doctors before or since the pandemic.

## Introduction

 Electronic health records (EHR) are commonplace in many healthcare settings and are often indispensable in care delivery. EHR replace paper-based medical charts to document patient clinical history, disease progression and medications, facilitate billing and enable communication with other healthcare providers.^1^,^3^ However, their implementation in the preceding decades has been fraught with difficulty.^3^,^6^ In the English NHS, various national policies and local initiatives have hastened the technology’s introduction into clinical settings.^4 7^ However, this resulted in a patchwork of EHR systems being adopted, but with limited clinical data sharing capabilities between them.^47^,^9^ This contributed to considerable data fragmentation across various providers, suboptimal use of health data to improve overall care quality and a largely inefficient and frustrating care-seeking experience for patients.^8 10^

Interoperability can facilitate effective care coordination, clinical decision support and healthcare user satisfaction.^11^,^13^ The Healthcare Information and Management Systems Society (HIMSS) in the USA defined interoperability as *‘the ability of different information systems, devices and applications (systems) to access, exchange, integrate and cooperatively use data in a coordinated manner, within and across organizational, regional and national boundaries, to provide timely and seamless portability of information and optimize the health of individuals and populations globally’.*^11 14^ In the UK, NHS England has similarly defined interoperability as *‘the capability for people involved in the provision and receipt of care to interact and complete a task across software and organisational boundaries; and use equipment, systems, or products from different vendors, which operate together in a coordinated fashion, with minimal to no human intervention’*.^15^

Yet, despite recognising what interoperability is and the value it brings to healthcare settings, the inability to easily access, modify and share clinical information has been highlighted by many.^16^,^20^ For physicians and patients, poor EHR interoperability reportedly negatively impacted work productivity, created additional communication barriers between clinical teams, increased clinician burnout, necessitated time-consuming workarounds and compromised patient safety.^1213 16^,^18 21^ Poor interoperability was also noted to be detrimental to overall EHR data quality, as it contributed to patient data fragmentation.^7 17 24^

While research examining EHR implementation is extensive, studies focussing on the interoperability of EHR systems are comparatively scarce.^1925^,^27^ A 2018 Canadian study investigated what elements of interoperable EHR systems in emergency departments in Alberta were most useful to physicians. The authors found that most of the time spent on EHR pertained to reviewing patient information useful for clinical decision-making.^28^ Clinical settings using read-only interoperable EHR systems, which lacked built-in patient management or clinical decision support tools, contributed to EHR disuse even when compared with those still reliant on paper-based processes. Altogether, the authors concluded that the clinical impact of using interoperable EHR remained *‘poorly understood’*.^28^

Another study explored the experiences of EHR use in primary care and community-based behavioural health settings in the USA.^21^ The study highlighted that providers often resorted to various workarounds to compensate for a lack of EHR interoperability.^21^ These included the duplication of data entry and documentation, physically printing documents to scan or fax to share, relying on patients’ and clinicians’ recollection for information and using *‘freestanding’* tracking systems (eg, an Excel spreadsheet).^21^ Another American study explored the facilitators and barriers to interoperability through interviews with hospital leaders, primary care providers, behavioural health providers and regional health information exchange networks.^25^ The authors found that the expansion of health information technology (HIT) applications to suit differing needs but are otherwise not interoperable can contribute to data fragmentation and information overload for end-users.^25^ The resulting siloing of clinical information was recognised to potentially jeopardise the safety, quality and efficiency of care.^25^

To the best of our knowledge, few have quantified and mapped the prevalence of poor EHR interoperability and its perceived effects on quality and safety from physician perspectives.^20 29^ In particular, none have completed or attempted to do so in the UK NHS context.^20 29^ This study aimed to understand how physicians perceive a lack of interoperability impacts their day-to-day clinical activities, affects patient safety and changes the productivity of their work.

### Aims

The overall aims of this study are threefold:

1. To capture the perceptions of physicians regarding the current state of EHR interoperability.
2. To investigate how a lack of interoperability affects patient care and patient safety.
3. To estimate the effect of a lack of EHR interoperability on care efficiency and costs.

## Methods

### Study population

Participants were NHS doctors practising in publicly funded facilities at different stages of training, ranging from trainees to consultants across both hospital and community-based settings. This included a wide range of specialties such as internal medicine, surgery, emergency medicine, anaesthesia, general practice, paediatrics and psychiatry.

### Sampling

The calculated minimum sample size required for this study was 287 respondents. This was determined using Cochran’s formula based on a desired 95% CI, 5% margin of error and an anticipated response rate of 25%, for a population size of 67 066 physicians currently working in the four main specialties most commonly associated with providing care for patients with chronic conditions (ie, internal medicine, surgery, emergency medicine, general practice (GP)) currently employed in the NHS, reported as of September 2020.^30^ A more conservative anticipated response rate was selected due to the ongoing COVID-19 pandemic at the time this study was being conducted. However, this value is still consistent with similar estimates found in the literature regarding the use of web-based surveys.^31^,^34^ Convenience sampling and snowballing techniques were used.

### Participant recruitment

The research team emailed existing contacts at Health Education England deaneries and other relevant institutions (eg, Royal College of General Practitioners) with a request to circulate the study advertisements widely via e-mail with members in their immediate clinical networks who met the inclusion criteria, as well as to personal contacts in other clinical settings. Study advertisements were circulated among trainee physicians by local heads of departments or trainee representatives who agreed to advertise the study in their healthcare facilities and via electronic newsletters. Reminders via e-mail and newsletters were sent out approximately every fortnight during the data collection period. No financial compensation was provided to participants. No follow-up assessments were held after data collection ended. Data collection lasted from June to October 2021.

### Description of questionnaire

The questionnaire comprised 42 questions and was available in English only (online supplemental file 1). The content was developed based on the existing literature and feedback from frontline NHS physicians.^2135^,^37^ This was organised into four sections:

- Part 1: basic participant demographic information.
- Part 2: physician EHR usage experience.
- Part 3: implications of EHR interoperability on patient safety and clinical care.
- Part 4: costs of healthcare resources accrued due to poor EHR interoperability.

The survey was piloted with four doctors (one GP, two surgical registrars and one internal medicine trainee) and iteratively refined. The survey and data collected were securely hosted and stored on Qualtrics, a web-based, password-protected survey platform licensed for use at Imperial College London.^38 39^

### Data analysis

Descriptive statistics were calculated for survey respondents, with cross-tabulations of respondent characteristics against survey responses also performed. Response rates per question were calculated, and the characteristics of respondents to each question were examined to identify any bias due to attrition over the survey course.

For questions exploring redundant diagnostic investigations performed, responses to individual types of tests (eg, FBC, urine dipstick, X-ray) were aggregated into *‘investigation-type’* categories (eg, blood-based, urine-based, radiological). An aggregated list of investigations is provided in online supplemental file 2, table A. When grouping responses within each *‘investigation-type’*, the most frequent response for any constituent test was used to represent the maximum number of tests conducted per category.

Two-tailed χ^2^ tests were performed to identify the relationship between participant characteristics of those who started the survey and completed it, with those participants who started the survey but did not complete the survey. All statistical analyses were performed using Stata 13.1.

## Results

### Participant characteristics

A total of 636 NHS doctors participated in the survey, of which 218 (34.3%) have completed it in its entirety. An overall response rate was not determinable due to the nature of the convenience sampling and snowballing recruitment approach taken. A full description of the respondents is provided in table 1. Of those who responded, 282 (47.4%) were females, and 224 (37.2%) were aged between 30 and 39. London (n=155, 28%) and Northwest England (n=131, 23.7%) received the greatest number of responses, and 48.1% were working in academic hospital settings. GPs comprised the largest clinical training group among participants (n=266, 44.1%).

**Table 1:** Characteristics of study participants. Percentages expressed in the ‘Missing’ rows are based on the total number of study participants.

Characteristics	N	Out of total number of participants (%)	Of those who responded (%)
Gender			
Female	282	44.3%	47.4%
Male	313	49.2%	52.6%
Missing	41	6.5%	
Age band (years)			
Under 30	122	19.2%	20.2%
30–39	224	35.2%	37.2%
40–49	145	22.8%	24.1%
50–59	84	13.2%	13.9%
60+	28	4.4%	4.6%
Missing	33	5.2%	
Clinical role			
Foundation year (FY1-2)	59	9.3%	9.8%
Senior house officers (ST1-2)	98	15.4%	16.3%
Registrar/Senior registrar (ST3+)	180	28.3%	29.9%
Consultant/GP	266	41.8%	44.1%
Missing	33	5.2%	
Location of practice			
East of England	52	8.2%	9.4%
London	155	24.4%	28%
Midlands	54	8.5%	9.8%
North East and Yorkshire	91	14.3%	16.4%
North West	131	20.6%	23.7%
South East	30	4.7%	5.4%
South West	41	6.5%	7.4%
Missing	82	12.9%	
Medical specialty training			
Internal medicine and subspecialties	226	35.5%	37.7%
Surgery and subspecialties	123	19.3%	20.5%
A&E	56	8.8%	9.3%
Anaesthesia	48	7.6%	8%
GP/family medicine	50	7.9%	8.3%
Paediatrics	43	6.8%	7.2%
Psychiatry	41	6.5%	6.8%
Other	13	2%	2.2%
Missing	36	5.7%	
Type of healthcare facility			
Academic/teaching hospital	290	45.6%	48.1%
District general/community hospital	244	38.4%	40.5%
GP practice	42	6.6%	7%
Other	27	4.3%	4.5%
Missing	33	5.2%	
Number of organisations interacting with			
None	7	1.1%	1.5%
1–2	58	9.1%	12%
3–4	144	22.6%	29.8%
5+	275	43.2%	56.8%
Missing	152	23.9%	
Received formal EHR training			
Yes	256	40.3%	56.5%
No	197	30%	43.5%
Missing	183	28.8%	
Self-reported EHR proficiency level			
Beginner	23	3.62%	5%
Moderate	235	37%	50.7%
Advanced	167	26.3%	36%
Expert	39	6.1%	8.4%
Missing	172	27%	
Years of experience using EHR			
Less than 1 year	25	3.9%	5.4%
1–2 years	101	16%	22%
3–5 years	138	21.7%	30%
6–10 years	115	18.1%	25%
More than 10 years	83	13.1%	18%
Number of clinical sessions per week			
None	1	0.2%	2.3%
1–2	7	1.1%	16.3%
3–4	10	1.6%	23.3%
5+	25	3.9%	58.1%
Missing	593	93.2%	
Frequency of EHR use			
Less than once a month	2	0.3%	0.4%
At least once a month	7	1.1%	1.5%
At least once a week	31	4.9%	6.7%
Every day	424	66.7%	91.4%
Missing	172	27%	

EHR, electronic health records; GP, general practice.

### Perceptions of healthcare providers regarding the current state of EHR interoperability

#### Interoperability-related EHR functions currently available and in use

Recognising that what interoperability-related EHR functions (ie, functions which involve the input and transfer of health data between two or more EHR elements or users) are available to NHS doctors may not necessarily align with what are routinely used, participants were asked to identify which functions were present and in common use at their workplace (online supplemental file 2, table C). The three most commonly available functions highlighted by respondents were (1) retrieval of patient’s previous health information (n=429/461, 93.1%), (2) inputting orders for investigations and medications (n=411/461, 89.2%) and (3) planning patient disposition and discharges (n=329/461, 71.4%). The most frequently used functions reported were (1) retrieval of patient’s previous health information (n=291/461, 63.1%), (2) inputting orders for investigations and medications (n=261/461, 56.6%) and (3) communicating with other healthcare professionals (n=163/461, 35.4%).

#### Directionality of interoperability present in existing EHR systems

Most respondents reported that they can view clinical information inputted by other healthcare providers within their own healthcare setting or facility (n=418/461, 90.7%) (online supplemental file 2, table D). However, visibility of clinical information outside of their immediate healthcare setting (n=175/460, 38%) and the ability for external healthcare providers to see their inputted data (n=74/457, 16.2%) were markedly lower.

Most respondents stated that they cannot edit clinical information within participants’ healthcare settings (n=225/460, 48.9%) and that from external healthcare providers (n=381/452, 84.3%). Conversely, clinical information in the participant’s hospital or clinic is typically not viewable and editable by most external healthcare providers (n=309/456, 67.8%). Of note was the increase in the number of ‘I do not know’ responses corresponding with EHR interactions of increasing complexity.

#### Impact of EHR interoperability on patient care

Most participants (n=396/413, 95.9%) reported that they have experienced difficulties retrieving clinical information from EHR systems (online supplemental file 2, table E). Of these, a quarter (n=100, 24.2%) stated that this occurred most of the time or always. Most respondents reported that poor EHR interoperability negatively impacted their day-to-day clinical workflow (n=386/412, 93.7%), of which 127 (n=127, 30.8%) stated that this occurred most of the time or always. Similarly, 81.5% (n=335/411) of respondents reported that they felt poor EHR interoperability posed a potential risk to patient safety (eg, incomplete medical histories, inaccurate medication lists, missing drug allergies), with 19.2% (n=79) describing it as a risk most of the time or always. Most clinicians reported that poor EHR interoperability negatively affected their ability to share clinical information with other healthcare professionals (n=371/411, 90.3%). Similarly, 352 of 409 respondents (86.1%) described it as being detrimental to their communication with patients and caregivers.

#### Impact on EHR data visibility and tasks

Three problems associated with data visibility and completion of EHR tasks most frequently identified by respondents were: (1) difficulty retrieving patient information available in another healthcare (n=300, 83.6%), (2) difficulty accessing patient information even when you know that information is available locally within the system (n=217, 60.5%) and (3) difficulty conveying clinical information for another healthcare professional (n=216, 60.2%) (online supplemental file 2, table F).

### How interoperability affects patient care safety

**Figure 1:**
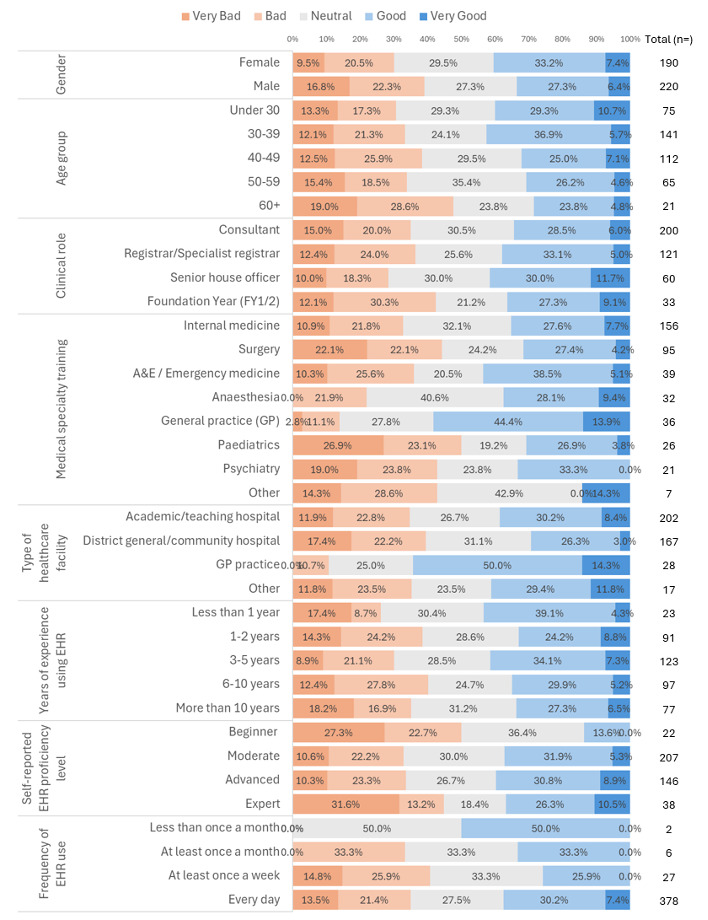
Breakdown of characteristics of doctors reporting experiences of electronic health records (EHR) interoperability at their workplace.

When asked to rate their overall experience of EHR and interoperability in the current workplace, participants reported largely positive (‘Good’, n=124, 30%) or neutral experiences (‘Neutral’, n=117, 28.3%). ‘Bad’ (n=90, 21.7%), ‘Very bad’ (n=55, 13.3%) and ‘Very good’ (n=28, 6.8%) comprised the remainder of the responses received. A large proportion of negative experiences (Very bad and Bad) was reported by those having a lower self-reported EHR proficiency. Conversely, GPs and doctors practising in non-secondary care centres tended to report comparatively positive experiences with EHR interoperability in contrast to other specialties. The reported experiences cross-tabulated against the various participant characteristics are shown in figure 1.

**Figure 2:**
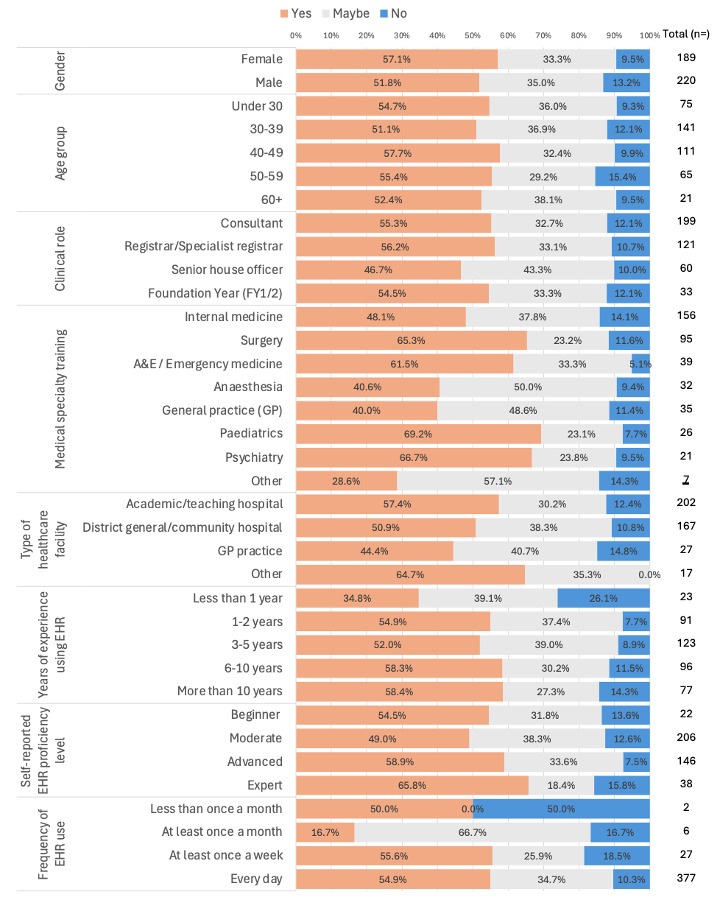
Doctor perceptions of whether limited electronic health records (EHR) interoperability poses a patient safety risk.

Out of 413 responses received, most participants (n=366, 88.6%) indicated they believed that the lack of EHR interoperability does or may pose a risk to patient safety, with 224 (52.2%) providing a ‘Yes’ response. The perceived risk to patient safety is most prevalent among doctors in specialties based in secondary care centres (eg, surgery, A&E and paediatrics) and those who have had less than a year’s experience using EHR. For a breakdown of the responses by participant characteristics, please see figure 2.

#### Mapping the impact of the lack of EHR interoperability along the pathway of care

To identify where along the clinical care pathway the lack of EHR interoperability typically occurs, participants were asked when they find it most impeding to their clinical work during a routine clinical shift. Out of the total number of study participants, only 310 (48.7%) completed this question (online supplemental file 2, table G). The most common response was when receiving patients from a secondary or tertiary healthcare facility (n=224, 72.3%), followed by medication reconciliation (n=178, 57.4%) and transferring patients to another healthcare facility (n=175, 56.5%).

### Care inefficiencies and associated healthcare costs incurred

To gain a better understanding of the impact on physician productivity resulting from poor EHR interoperability, participants were asked whether they felt poor EHR interoperability has negatively impacted their workflow in terms of (1) repeat diagnostic investigations, (2) prolonged length of stay (PLOS) in the hospital and (3) prolonged clinic preparation and consultation times. Participants who responded ‘Yes’ were then asked to quantify their response for each category.

#### Repeat diagnostic investigations

For repeat diagnostic investigations, participant responses were aggregated into investigation-type categories (online supplemental file 2, table H). Responses for each investigation type are detailed in online supplemental file 2, table B. Infectious panels were reported to be the most repeated on a daily basis due to poor interoperability (n=194, 73.5%). Radiological investigations were the second most repeated, with physicians reportedly needing to do so once per week (n=125, 43.6%). This is followed by bloodwork (n=114, 39.5%) and urine-based investigations (n=87, 32.6%), both of which require repeating typically on a weekly basis.

#### Prolonged length of stay in hospital

When questioned as to whether problems sharing or retrieving clinical information via EHR with limited interoperability resulted in keeping patients in the hospital longer, 143 out of 222 (64.4%) respondents responded ‘Yes’. When asked to quantify the extent of delay for patients where limited EHR interoperability contributed to a problem, 105 (42.9%) participants reported that issues with EHR interoperability often resulted in several hours of delays in discharging patients, 53 (21.6%) reported that it typically caused one-night additional stay in hospital, 52 (21.2%) saw no delays caused and 35 (13.2%) caused two+ nights of additional hospital stay.

Delays of several hours were reported most frequently from surgical subspecialties (n=42, 51.9%), medicine subspecialties (n=21, 29.2%) and A&E (n=17, 51.5%) (online supplemental file 2, table I). Delays causing one additional night stay were most reported by medicine subspecialties (n=25, 34.7%), followed by A&E (n=9, 27.3%) and surgical subspecialties (n=9, 11.1%).

#### Prolonged clinic consultation times

Most participants stated that interoperability problems have necessitated extra time during routine clinic consultations (online supplemental file 2, table J), both when preparing for (n=209, 95.9%) as well as during consultations themselves (n=211, 96.8%). Most participants required between an extra 15–30 min of preparation time (n=72, 33%) and another 15–30 min of consultation time during a routine day of clinic consultations (n=71, 32.6%).

## Discussion

### Summary of principal results

This survey of 636 NHS doctors in England provides an important insight into how EHR interoperability, or a lack thereof, affects their day-to-day practice.

EHR are widely used by clinicians to retrieve clinical information about their patients and also to input information into clinical systems, including for the ordering of investigations and medications. Further, a significant minority of doctors report using EHR to communicate with other healthcare colleagues.

While most doctors are able to view patient information in their own hospital EHR (91%), far fewer can edit this information (43%), likely reflecting constraints on editing medical records written by others. Many respondents reported being able to view information from other healthcare organisations (38%) but did not believe other organisations were able to view the information they had inputted (16%). This finding potentially indicates either a lack of reciprocity in EHR interoperability or a lack of awareness of the interoperability capabilities of providers with whom clinicians regularly share patients. This latter point is supported by increasing numbers of ‘don’t know’ responses to questions pertaining to what was possible at other providers than the respondent’s own. When attempting to map out where issues with limited EHR interoperability typically occur along a patient’s clinical pathway, participants largely highlighted this manifesting during the transition of care between two separate NHS settings, as well as when doctors are completing medication reconciliation.

Almost all participants reported experiencing some form of difficulty in accessing clinical information due to poor EHR interoperability. Unsurprisingly, many stated that this was detrimental to their clinical workflow and impaired their communication with patients and other healthcare providers. In terms of current experiences of interoperability at their workplace, negative views were more prevalent among doctors who practised in specialties based in secondary care settings as well as those with less than 1 year of EHR experience and lower self-reported EHR proficiency levels. Regarding the perceived impact on patient safety overall, most doctors reported that the limited EHR interoperability they routinely experienced does pose some form of risk (eg, incomplete medication/allergy lists, missing clinical letters/documentation). This finding consistently outnumbered those who did not share this view across all participant characteristics.

However, the impact of lack of EHR interoperability on care efficiency was mixed. Infection panels were repeated by many respondents on a daily basis, with other redundant investigations being done so typically weekly. Most reported that poor EHR interoperability led to PLOS and prolonged consultation times. However, responses quantifying delays varied between specialties (eg, surgeons tended to report shorter delays than internists).

### Comparison with prior work

To better understand EHR systems used across the UK, Warren *et al* used national-level administrative data to explore the spatial distribution of EHR systems in relation to their use when patients seek care between NHS trusts.^7^ The authors found that although a total of 21 different EHR systems were in use and three vendors’ systems made up the majority, most hospitals did not have robust standardised means of electronic clinical data sharing. Even when patients sought care between two trusts that used EHR from the same vendor, only 0.6% of patients were able to take advantage of the greater interoperability possible.^7^ The authors highlighted that while 20 pairs of trusts were found to routinely share the same cohort of patients, only one pair used EHR systems, which were from the same vendor and allowed for some form of interoperability.^7^ Though this study was valuable in identifying macro-level trends, the secondary data used did not allow for more granular insights into how poor interoperability materialises along a patient’s care pathway.

Investigating the amount of additional healthcare expenditure because of poor interoperability has been explored. For example, Stewart *et al* examined whether poor interoperability was associated with increases in duplicate medical tests conducted in patients with congenital heart disease being shared between two nearby hospitals with no EHR interoperability in Boston, Massachusetts.^40^ Of the 27 patient records that showed duplicate investigations performed, 17 were not clinically indicated and half had more than one test duplicated.^40^ The majority of these were conducted during admission from outpatient clinics, with authors speculating that this was likely due to the delays or difficulties encountered during the transfer of test results.^40^ An estimated US$ 1255 was, thus, accrued to all the study patients because of duplicated investigations (US$ 14.56 per patient). While this sum was modest, the savings from minimising duplicative efforts is likely substantial.^40^ However, this study’s generalisability is limited as it pertained only to a highly specialised patient group and used a narrow definition of *‘duplicate test’*.^40^

In a similar attempt to explore the potential cost benefits resulting from increased interoperability, Meyer *et al* measured the difference in perceived and actual time spent by nurses completing clinical task orders when using EHR systems with and without interoperability in Geneva-based hospitals.^41^ An EHR system used in one hospital without interoperability was compared with one introduced at another hospital, which had interoperability features available. The authors found that interoperability between the various systems saved on average 26 s per order entry, allowing the systems to be used approximately three times as fast.^41^ When extrapolated with the wages for nurses at approximately US$ 0.73 per minute and roughly 20 000 laboratory orders generated monthly, the authors estimated that incorporating interoperability between various HIT systems contributed on average, US$ 6325 of work-time equivalent savings per month.^41^

### Strengths and limitations

This is the first study exploring challenges posed to NHS physicians when using EHR with varying levels of interoperability, ascertaining where interoperability-related issues arise on clinical pathways and examining the impact on productivity in the NHS. This study comprised a relatively large sample size, received responses from physicians across England and included participants from hospital and community-based settings. The survey itself is methodologically robust, with the content derived from a systematic review of the wider literature and subsequently piloted with four physicians of varying clinical experience from three different specialties. Reporting of the study design, analytical approach and findings is done so in accordance with the Consensus-Based Checklist for Reporting of Survey Studies (CROSS).^42^

Limitations are largely inherent to survey studies themselves. Given that the findings are self-reported, there is a risk of self-selection and recall bias. While stratified purposive sampling was initially planned to ensure a proportionate representation of physicians from different backgrounds, convenience sampling was eventually used due to pragmatic considerations during the ongoing pandemic circumstances. In addition, doctors from specialties who did not solely care for patients with chronic conditions (eg, A&E) were ultimately included in the study. The authors recognised that while this practical accommodation may have negatively impacted the accuracy of the initial sampling calculations, it was thought that capturing the experiences of doctors in acute care settings, particularly during the unfolding pandemic, would be undoubtedly valuable nonetheless. The survey being performed during COVID-19 may have also elicited views regarding interoperability issues, which may not be representative of routine EHR usage experienced by NHS doctors before or after the pandemic. Another limitation was the relatively high attrition rate among certain subgroups to finishing the study. For example, the small number of responses from Foundation Year doctors at the beginning of the survey (n=59), dropping to a handful by the end (n=8), made an analysis of their responses a notable challenge. Given this subgroup tends to interact with EHR more senior staff, the lack of responses from them contributes to a loss of granularity in the overall data captured.

### Implications for practice, policy and further research

NHS physicians largely reaffirmed that poor EHR interoperability is a widespread problem across the health system, and thus negatively impacts their ability to deliver safe and efficient care.

GPs were the only standout group among the study cohort who reported comparatively positive experiences with EHR interoperability. This is potentially due to GPs generally having greater control over the EHR systems in use at their clinics and the less frequent need to interact with other clinical teams to facilitate routine patient care. However, given the high number of responses highlighting interoperability-related issues when receiving patients from secondary care (n=224), this suggests that problems with inter-organisational EHR interoperability are more pronounced than those within primary care settings themselves. Together, this may have contributed to mitigating some of the overall negative experiences perceived by GPs compared with doctors in secondary care.

The variation in negative views from doctors across the levels of self-reported EHR proficiency also points to a potential gap in EHR skills for young trainees entering their first clinical workplace. Once junior doctors are familiarised with the EHR available, the slight reduction of negative views suggests that greater experience may have helped them overcome some of the initial perceived system shortcomings (eg, workflow inconveniences). Those who eventually become *‘experts’* with their EHR are likely to be more vocal about the negative experiences surrounding interoperability issues routinely encountered. This trend is similar regarding the perceived risk to patient safety—those who have had less time using EHR have yet to recognise it as a threat to patient safety compared with more seasoned users. Nonetheless, this did not alter the prevailing perception among most doctors that current levels of EHR interoperability remain inherently unsafe.

Regarding the impact on clinical tasks, responses largely indicated the inability to view the results of recently conducted investigations and difficulty in communicating with other health professionals. The mismatch in responses quantifying the severity of interoperability-related challenges vs the perception of risks to care might be explained by limited interoperability causing poor end-user experiences, making it easier for errors to occur and indirectly causing harm.

Deriving an estimate of the impact of poor interoperability on productivity proved especially challenging. Nonetheless, respondents were able to highlight some notable areas of concern. For example, physicians reported that radiological investigations were the second most repeated type of investigation due to poor interoperability, with this occurring typically once a week. With investigations such as MRIs having a unit cost averaging £161.54, these additional investigations have the potential to incur more significant costs when extrapolated across the NHS.^43 44^ While most participants noted that interoperability-related discharge delays were common, surgical subspecialties typically kept patients for several more hours, while medical subspecialties reported longer delays, typically one additional night and up to 2–3 nights. Costs of delays lasting *‘several hours’* are difficult to establish given the imprecise definition, the opportunity costs based on bed location (eg, A&E vs wards) and the professionals involved. For reference, a non-elective short stay (ie, 2 days or less) is estimated to cost an average of £801 per unit.^43^

Recommendations to mitigate some of these challenges are gradually becoming available. The NHS Interoperability Toolkit, first released in 2010 and recently updated in March 2023, comprises a collection of frameworks, guidance material and technical reference documentation to streamline validation processes, lower costs and provide set interoperability standards for vendors.^45 46^ Policy-based approaches, such as those suggested by Zhang *et al,* proposed having regulatory bodies (eg, Care Quality Commission and NHSX) promote, enforce and push for systemic changes, which have proven difficult to do with the currently fragmented HIT procurement strategies between trusts and the lack of business incentives for system vendors to do so themselves.^10^ While regulatory bodies do not have the authority to dictate HIT policies directly, greater regulatory involvement would give a stronger sense of collective strategic direction in realising a more integrated network of EHR.

Future research should capture the perceptions of EHR interoperability from other healthcare workers (eg, nurses, pharmacists, allied health workers), ascertain how problems arise along clinical pathways and measure the impact on patient care and safety using standardised key performance indicators. Given that other NHS healthcare professionals may be similarly affected by limited EHR interoperability, there is a clear imperative to explore these perspectives, particularly through the employment of qualitative methodologies such as semi-structured interviews. When conducted alongside quantitative methodologies, this would allow for a deeper and more comprehensive interrogation of the experiences and specific challenges encountered in clinical practice. Future studies designed with larger sample sizes, more robust sampling strategies and powered to allow for comparisons of reported levels of EHR interoperability between different physician characteristics to be made (eg, between specialties, hospital vs community-based settings) would likely be an invaluable next step. Another under-explored aspect worth investigating is the interoperability challenge encountered by public and private NHS healthcare providers. Research examining EHR user experience, technical aspects of interoperability or tangential areas such as implementation sciences and economic analyses can advance the understanding of the value posed by interoperable EHR.^47^

## Conclusion

In the English NHS, EHR interoperability is minimal, sporadic and poses a multitude of practical workflow, communication and potential safety challenges for frontline NHS doctors. While there is a broad consensus regarding its detrimental effects, the perceived impact of poor interoperability is not felt uniformly across the NHS. Certain physician characteristics such as specialty or years of experience using EHR influence their perception of interoperability and how they believe it could impact patient care. Poor interoperability was reported to necessitate repeat diagnostics, lengthen hospital stays and prolong clinic consultations, though the related burdens were not perceived to be severe. However, when extrapolated across the whole of the NHS, these seemingly less significant hindrances would likely culminate in substantial inefficiencies and costs.

This study illuminates the perceptions of poor interoperability in practice from users themselves. As the first of its kind in the UK attempting to *‘take the pulse’* of how robust health information exchanges are implemented across various healthcare settings, this study sets the stage for future efforts to more thoroughly investigate the extent of the problem that poor EHR interoperability poses. Future efforts at overhauling EHR interoperability must incorporate technical as well as policy-based solutions, which closely reflect the practical needs, concerns and feedback of end-users.

## Supplementary material

10.1136/bmjopen-2024-096669 online supplemental file 1

10.1136/bmjopen-2024-096669 online supplemental file 2

## Data Availability

Data are available upon reasonable request.
