# Supplementary material for: Physician experiences of electronic health record interoperability and its practical impact on care delivery in the English NHS: a cross-sectional survey study
Source: BMJ Open. 2025 Jun 10;15(6):e096669. doi: 10.1136/bmjopen-2024-096669 (PMC12161308; doi:10.1136/bmjopen-2024-096669)
Supplement: online supplemental file 2 [file bmjopen-15-6-s002.docx]

## Supplement 2: Data tables A-J

Table A: Repeat investigation categories examined.

| **Categories** | **Investigations** |
| --- | --- |
| Bloodwork | Baseline panels (e.g., FBC, U&E)  Cardiac profile (e.g., troponin)  Thyroid panel (e.g., TFT)  Liver panel (e.g., LFT)  Lipid profile (e.g., LDL/HDL)  Inflammatory markers (e.g., ESR/CRP)  Blood gases ABG/VBG |
| Urine-based | Urine dipstick  Urinalysis  Infectious panels Urine culture  Blood culture |
| Radiology | Ultrasound  Plain film/X-rays  CT scans  MRI |
| Miscellaneous | Point-of-care tests (e.g., Group A Strep, RSV)  βHCG pregnancy test  Other investigations |
| Non-applicable | Non-applicable |

Table B: Repeat diagnostic investigations performed because of poor EHR interoperability (non-aggregated figures) from total number of responses.

|  | **Daily**  **n (%)** | **4-6 times a week n (%)** | **2-3 times a week n (%)** | **Once a week n (%)** | **Never n (%)** |
| --- | --- | --- | --- | --- | --- |
| Baseline panels (i.e., FBC, U&E) | 12 (4.29%) | 12 (4.29%) | 32 (11.43%) | 97 (34.64%) | 127 (45.36%) |
| Blood gas (i.e., ABG, VBG) | 3 (1.10%) | 3 (1.10%) | 11 (4.04%) | 48 (17.65%) | 207 (76.10%) |
| Cardiac profile (i.e., troponins) | 1 (0.38%) | 1 (0.38%) | 10 (1.57%) | 37 (13.96%) | 216 (81.51%) |
| Thyroid panel (i.e., TFT) | 4 (1.48%) | 2 (0.74%) | 9 (3.33%) | 68 (25.19%) | 187 (69.26%) |
| Liver panel (i.e., LFT) | 4 (1.49%) | 1 (0.37%) | 15 (5.58%) | 80 (29.74%) | 169 (62.83%) |
| Lipid profile (i.e., LDL/HDL) | 4 (3.37%) | 1 (0.37%) | 9 (3.37%) | 48 (17.98%) | 205 (76.78%) |
| Inflammatory markers (i.e., ESR/CRP) | 8 (2.97%) | 6 (2.23%) | 13 (4.83%) | 84 (31.23%) | 158 (58.74%) |
| Blood culture | 2 (0.76%) | 0 (0.00%) | 5 (1.91%) | 64 (24.43%) | 191 (72.90%) |
| Urine dipstick | 12 (4.62%) | 11 (4.23%) | 40 (15.38%) | 61 (23.46%) | 136 (52.31%) |
| Urinalysis | 11 (4.21%) | 9 (3.45%) | 25 (9.58%) | 68 (26.05%) | 148 (56.70%) |
| Urine culture | 5 (1.92%) | 5 (1.92%) | 16 (6.13%) | 62 (23.75%) | 173 (66.28%) |
| βHCG pregnancy test | 8 (3.07%) | 3 (1.15%) | 13 (4.98%) | 38 (14.56%) | 199 (76.25%) |
| Point-of-care tests | 4 (1.57%) | 4 (1.57%) | 7 (2.75%) | 32 (12.55%) | 208 (81.57%) |
| Ultrasound | 6 (2.21%) | 3 (1.10%) | 18 (6.62%) | 75 (27.57%) | 170 (62.50%) |
| Plain film/X-rays | 5 (1.85%) | 4 (1.48%) | 13 (4.81%) | 93 (34.44%) | 155 (57.41%) |
| CT scans | 4 (1.46%) | 4 (1.46%) | 12 (4.38%) | 82 (29.93%) | 172 (62.77%) |
| MRI | 4 (1.50%) | 3 (1.12%) | 7 (2.62%) | 66 (24.72%) | 187 (70.04%) |
| Other investigations | 5 (4.07%) | 2 (1.63%) | 4 (3.25%) | 24 (19.51%) | 88 (71.54%) |
| Not applicable | 13 (11.50%) | 1 (0.88%) | 2 (1.77%) | 5 (4.42%) | 92 (81.42%) |

Table C: EHR functions commonly available and in use by NHS doctors^[[1]](#footnote-1)^

|  | **My available EHR can perform these functions, n (%)** | **I often perform these functions using my EHR, n (%)** |
| --- | --- | --- |
| Hospital administrative processes and reporting | 327 (70.9%) | 139 (30.2%) |
| Input orders for investigations/medications | 411 (89.2%) | 261 (56.6%) |
| Public health surveillance and reporting | 129 (28%) | 56 (12.1%) |
| Planning patient disposition/discharges | 329 (71.4%) | 154 (33.4%) |
| Aid in clinical decision-making | 282 (61.2%) | 157 (34.1%) |
| Retrieve patient’s previous health information | 429 (93.1%) | 291 (63.1%) |
| Communicate with other healthcare professionals | 299 (64.9%) | 163 (35.4%) |
| Communicate with and support patients | 162 (35.1%) | 82 (17.9%) |

Table D: Directionality of interoperability of existing EHR systems.

|  | **Yes, n (%)** | **No, n (%)** | **I do not know, n (%)** |
| --- | --- | --- | --- |
| I can SEE clinical information inputted by other healthcare providers WITHIN my healthcare setting. (n=461) | 418 (90.7%) | 36 (7.8%) | 7 (1.5%) |
| I can SEE clinical information inputted by healthcare providers FROM EXTERNAL hospitals/clinics. (n=460) | 175 (38%) | 251 (54.6%) | 34 (7.4%) |
| I can both SEE & EDIT clinical information inputted by other healthcare providers WITHIN my healthcare setting. (n=460) | 197 (42.8%) | 225 (48.9%) | 38 (8.3%) |
| I can both SEE & EDIT clinical information inputted by healthcare providers FROM EXTERNAL hospitals/clinics. (n=452) | 23 (5.1%) | 381 (84.3%) | 48 (10.6%) |
| Healthcare providers FROM EXTERNAL hospitals/clinics, can SEE all the clinical information I have inputted. (n=457) | 74 (16.2%) | 259 (56.7%) | 124 (27.1%) |
| Healthcare providers FROM EXTERNAL hospitals/clinics, can both SEE & EDIT the clinical information I have inputted. (n=456) | 16 (3.5%) | 309 (67.8%) | 131 (28.7%) |

Table E: Impact of interoperability on patient care, safety, and clinical workflow.

|  | **Always, n (%)** | **Most of the time, n (%)** | **About half the time, n (%)** | **Sometimes, n (%)** | **Never, n (%)** |
| --- | --- | --- | --- | --- | --- |
| Difficulty accessing and retrieving clinical information through the EHR systems currently in use. (n= 413) | 38 (9.2%) | 62 (15%) | 64 (15.5%) | 232 (56.2%) | 17 (4.1%) |
| Difficulty with accessing and retrieving this clinical information negatively affects my day-to-day clinical workflow. (n=412) | 55 (13.4%) | 72 (17.5%) | 56 (13.6%) | 203 (49.3%) | 26 (6.3%) |
| Difficulty with accessing and retrieving this clinical information poses a potential risk to patient safety during my routine shifts in the hospital/clinic. (n=411) | 30 (7.3%) | 49 (11.9%) | 27 (6.6%) | 229 (55.7%) | 76 (18.5%) |
| Difficulty with accessing and retrieving this clinical information negatively impacts my ability to share clinical information with other healthcare professionals. (n=411) | 46 (11.2%) | 72 (17.5%) | 55 (13.4%) | 198 (48.2%) | 40 (9.7%) |
| Difficulty with accessing and retrieving this clinical information negatively impacts my ability to share clinical information with my patients and/or their caregivers. (n=409) | 47 (11.5%) | 56 (13.7%) | 46 (11.3%) | 203 (49.6%) | 57 (13.9%) |
| Thinking about your patients’ expectations regarding the accessibility of their health records, do you feel that the EHR systems you currently use allow you to meet these expectations? (n=408) | 21 (5.2%) | 76 (18.6%) | 70 (17.2%) | 165 (40.4%) | 76 (18.6%) |

*Table F: Difficulties with EHR data availability and tasks due to poor interoperability* ^^[[2]](#footnote-2)^^

|  | **Yes, n (%)** |
| --- | --- |
| Do not know if the information is available in the EHR system or one that is connected to it. | 201 (56%) |
| Difficulty accessing patient information even when you know that information is available within the system | 217 (60.5%) |
| Difficulty retrieving patient information you know is available in another healthcare facility frequented by the patient | 300 (83.6%) |
| Difficulty following up on an order (e.g., test results) you inputted previously | 152 (42.3%) |
| Difficulty conveying clinical information for another healthcare professional | 216 (60.2%) |

Table G: When is the lack of interoperable EHRs most impeding to your clinical work during a routine clinical shift?

|  | **Yes, n (%)** | **No, n (%)** |
| --- | --- | --- |
| Admitting a new patient from the community | 152 (49%) | 158 (51%) |
| Receiving a patient from a secondary/tertiary healthcare facility | 224 (72.3%) | 86 (27.7%) |
| During handover of patients from other members within my immediate clinical team | 51 (16.5%) | 259 (83.5%) |
| During handover of a patient from another clinical team/clinician within my hospital | 71 (22.9%) | 239 (77.1%) |
| Following up on an order from another clinical team/clinician in my hospital | 102 (32.9%) | 208 (67.1%) |
| Discharging patient from my hospital back into the community | 102 (32.9%) | 208 (67.1%) |
| Transferring a patient from my hospital to another secondary/tertiary healthcare facility | 175 (56.5%) | 135 (43.6%) |
| Medication reconciliation | 178 (57.4%) | 132 (42.6%) |
| Other | 28 (9%) | 282 (91%) |

Table H: Repeat diagnostic investigations performed because of poor EHR interoperability as reported by 289 respondents who completed the question.

|  | **Bloodwork, n (%)** | **Blood Gases, n (%)** | **Urine-based, n (%)** | **Infectious Panel, n (%)** | **Radiology, n (%)** |
| --- | --- | --- | --- | --- | --- |
| Never | 108 (37.4%) | 207 (76.1%) | 125 (46.8%) | 21 (8%) | 123 (42.9%) |
| Once a week | 114 (39.5%) | 48 (17.7%) | 87 (32.6%) | 33 (12.5%) | 125 (43.6%) |
| 2-3 times a week | 35 (12.1%) | 11 (4%) | 30 (11.2%) | 13 (4.9%) | 25 (8.7%) |
| 4-6 times a week | 15 (5.2%) | 3 (1.1%) | 12 (4.5%) | 3 (1.1%) | 5 (1.7%) |
| Daily | 17 (5.9%) | 3 (1.1%) | 13 (4.9%) | 194 (73.5%) | 9 (3.1%) |

Table I: Cross tabulation of delays in hospital vs. specialty training. Percentages expressed are for values per row.

|  | **No delays, n (%)** | **Several hours delay, n (%)** | **1-night additional stay in hospital, n (%)** | **2+ nights additional stay in hospital, n (%)** |
| --- | --- | --- | --- | --- |
| Internal medicine | 6 (8.3%) | 21 (29.2%) | 25 (34.7%) | 20 (27.8%) |
| Surgery | 21 (25.9%) | 42 (51.9%) | 9 (11.1%) | 9 (11.1%) |
| A&E | 5 (15.2%) | 17 (51.5%) | 9 (27.3%) | 2 (6.1%) |
| Anaesthesia | 3 (18.8%) | 9 (56.3%) | 3 (18.8%) | 1 (6.3%) |
| Paediatrics | 8 (36.4%) | 11 (50%) | 2 (9.1%) | 1 (4.6%) |
| Psychiatry | 8 (53.3%) | 1 (6.7%) | 4 (26.7%) | 2 (13.3%) |
| Other | 1 (20%) | 3 (60%) | 1 (20%) | 0 (0%) |

Table J: Prolonged clinic times due to issues of EHR interoperability amongst participants who responded (preparation and during the consultation).

|  | **Preparing for consultations, n (%)** | **During consultations, n (%)** |
| --- | --- | --- |
| No extra time needed | 9 (4%) | 7 (3.2%) |
| Less than 5 minutes | 18 (8.3%) | 22 (10.1%) |
| 5-15 minutes | 56 (25.7%) | 64 (29.4%) |
| 15-30 minutes | 72 (33%) | 71 (32.6%) |
| 30-60 minutes | 45 (20.6%) | 43 (19.7%) |
| More than an hour | 18 (8.3%) | 11 (5.1%) |

1. It was not possible to differentiate between respondents who did not complete this question and those

   who completed the question but chose not to select any options. As such, the number of respondents who

   completed the next question in the survey (n=461), is taken as a denominator for the purpose of calculating

   percentages. [↑](#footnote-ref-1)
2. It was not possible to differentiate between respondents who did not complete this question and those

   who completed the question but chose not to select any options. As such, the number of respondents who

   completed the next question in the survey (n=359), is taken as a denominator for the purpose of calculating

   percentages. [↑](#footnote-ref-2)
