# Supplementary material for: Physician experiences of electronic health record interoperability and its practical impact on care delivery in the English NHS: a cross-sectional survey study
Source: BMJ Open. 2025 Jun 10;15(6):e096669. doi: 10.1136/bmjopen-2024-096669 (PMC12161308; doi:10.1136/bmjopen-2024-096669)
Supplement: Supplementary file 3 [file bmjopen-2024-096669.reviewer_comments.pdf]

PEER REVIEW HISTORY

BMJ Open publishes all reviews undertaken for accepted manuscripts. Reviewers are asked to complete a checklist review form and are provided with free text boxes to elaborate on their assessment. These free text comments are reproduced below.

ARTICLE DETAILS

Title (Provisional)

Physician Perceptions of Electronic Health Records Interoperability and its Practical Impact on Care Delivery in the English NHS: A Cross-sectional Survey Study

Authors

Li, Edmond; Lounsbury, Olivia; Hasnain, Mujtaba; Ashrafian, Hutan; Darzi, Ara; Neves, Ana Luisa; Clarke, Jonathan

VERSION 1 - REVIEW

**Reviewer**	**1**
**Name**	**Baji, Petra**
**Affiliation**	**University of Bristol**
**Date**	**22-May-2025**
**COI**	**None**

This is a well written manuscript on the conduct and the results of a national survey on physicians' perspectives of poor electronic health record (EHR) interoperability and its perceived impact on the quality and safety of care.

I have only a few minor comments and suggestions that may help understanding and clarity of the manuscript:

1. The concept of interoperability would benefit from a clearer definition early in the manuscript. Specifically, it would be helpful to describe what types of institutions or providers are meant to be interoperable—e.g., between hospitals, general practices, pharmacies… or across different NHS trusts or private sector. Clarifying this would set a more precise context for interpreting the findings.

2. Authors mention in the Introduction that "a few studies have quantified and mapped the prevalence of poor EHR interoperability and its perceived effects on quality and safety from physician perspectives" – It is not clear what was the perspective of previous studies mentioned in the Introduction. Maybe authors could add on the lack of studies on interoperability in the NHS/UK?

3. The authors mention in the Method/Sampling section that sample size was calculated based on the number of practitioners in the "four main specialties most commonly associated with providing care for patients with chronic conditions (i.e., internal medicine, surgery, emergency medicine, general practice (GP)) It was not clear if any specialties were excluded from the survey for not mainly providing care for patients with chronic conditions.

4. I wonder whether GPs were relatively under representative in the survey? The authors could expand discussion on the implications of that as they found different experiences for this subgroup. They could point out the differences in the use of EHR by GPs and hospital based specialists, which is already briefly mentioned in the Discussion.

5. It is unclear whether respondents from the private healthcare sector were included in the sample. Since interoperability challenges may differ between public and private providers, this should be explicitly stated and, if relevant, discussed.

6. Authors could provide examples what they mean by "safety risk for patients" and "posed a potential risk to patient safety"— for clarity and consistency.

7. Suggestion for further research: A qualitative component such as semi-structured interviews could enhance understanding of the more nuanced and critical interoperability issues that physicians face. Such methods would allow deeper exploration of the experiences and specific challenges encountered in clinical practice.

VERSION 1 - AUTHOR RESPONSE

Reviewer comments	Author responses
This is a well written manuscript on the conduct and the results of a national survey on physicians' perspectives of poor electronic health record (EHR) interoperability and its perceived impact on the quality and safety of care.	Thank you for taking the time to review our manuscript and your constructive feedback/comments.
1. The concept of interoperability would benefit from a clearer definition early in the manuscript. Specifically, it would be helpful to describe what types of institutions or providers are meant to be interoperable—e.g., between hospitals, general practices, pharmacies... or across different NHS trusts or private sector. Clarifying this would set a more precise context for interpreting the findings.	Thank you for this comment.<br><br>We certainly agree that providing definition early in the manuscript would aid in clarity for the reader. We have done this now and it reads as follows:<br><br>*'Interoperability can facilitate effective care coordination, clinical decision support, and healthcare user satisfaction [11–13]. The Healthcare Information and Management Systems Society (HIMSS) in the US defined interoperability as 'the ability of different*

	information systems, devices and applications (systems) to access, exchange, integrate and cooperatively use data in a coordinated manner, within and across organizational, regional and national boundaries, to provide timely and seamless portability of information and optimize the health of individuals and populations globally.' [14,15]. In the UK, NHS England has similarly defined interoperability as: 'the capability for people involved in the provision and receipt of care to interact and complete a task across software and organisational boundaries; and use equipment, systems, or products from different vendors, which operate together in a coordinated fashion, with minimal to no human intervention.' [16].' (Pg 3, lines 88-97)
2. Authors mention in the Introduction that "a few studies have quantified and mapped the prevalence of poor EHR interoperability and its perceived effects on quality and safety from physician perspectives" – It is not clear what was the perspective of previous studies mentioned in the Introduction. Maybe authors could add on the lack of studies on interoperability in the NHS/UK?	Thank you for this comment.<br><br>As per your recommendation, we have now added text to clearly highlight the lack of studies investigating interoperability in the NHS. It now reads as follows:<br><br>*To the best of our knowledge, few have quantified and mapped the prevalence of poor EHR interoperability and its perceived effects on quality and safety from physician perspectives [21,31]. In particular, none have completed or attempted to do so in the UK NHS context. This study aimed to understand how physicians perceive a lack of interoperability impacts their day-to-day clinical activities, affects patient safety and changes the productivity of their work. (Pg 4, lines 124-128)*
3. The authors mention in the Method/Sampling section that sample size was calculated based on the number of practitioners in the "four main specialties most commonly associated with providing care for patients with chronic conditions (i.e., internal medicine, surgery, emergency medicine, general practice (GP)) It was not clear if	Thank you for this comment.<br><br>The description of chronic conditions guided our original sample size calculations as it was initially envisaged that physicians who cared for patients with chronic conditions may be more predisposed to encountering issues with EHR interoperability.

any specialties were excluded from the survey for not mainly providing care for patients with chronic conditions.	However, participants who did not explicitly care for patients with chronic conditions were not excluded from the study. Although we appreciate this might impact the accuracy of our sample size calculations, we did not want to overlook capturing experiences of physicians in acute care settings, particularly during the COVID-19 pandemic, and therefore no non-chronic care physicians were excluded. Nonetheless, we can appreciate the point you raised here, and we have now added it in the limitations. The edited version of the manuscript now reads as follows:<br><br>*'While stratified purposive sampling was initially planned to ensure a proportionate representation of physicians from different backgrounds, convenience sampling was eventually used due to practical considerations during the ongoing pandemic circumstances. In addition, doctors from specialties who did not solely care for patients with chronic conditions (e.g., A&E) were ultimately included in the study. The authors recognised that while this practical accommodation may have negatively impacted the accuracy of the initial sampling calculations, it was thought that capturing the experiences of doctors in acute care settings, particularly during the unfolding pandemic, would be undoubtedly valuable nonetheless.' (Pg 13, lines 368-375)*
4. I wonder whether GPs were relatively under representative in the survey? The authors could expand discussion on the implications of that as they found different experiences for this subgroup. They could point out the differences in the use of EHR by GPs and hospital-based specialists, which is already briefly mentioned in the Discussion.	Thank you for your comment raising this point.<br><br>Our initial study design was not powered sufficiently for comparisons between the various specialties of doctors. We certainly agree there is room for future studies to tackle this specific shortcoming. This has now been added to the 'Future research' section as the following:

	'Future studies designed with larger sample sizes, more robust sampling strategies, and powered to allow for comparisons of reported levels of EHR interoperability between different physician characteristics to be made (e.g., between specialties, hospital vs community-based settings), would likely be an invaluable next step.' (Pg 15, lines 436-439)<br><br>Nonetheless, there appears a leaning towards physicians who are hospital-based vs those in the community. In terms of GPs specifically, this was likely due to recruitment methods employed: those specialties based in hospitals were easier to contact and send reminders to complete the survey compared to GPs practicing out in the community.
5. It is unclear whether respondents from the private healthcare sector were included in the sample. Since interoperability challenges may differ between public and private providers, this should be explicitly stated and, if relevant, discussed.	Thank you for your comment.<br><br>No private sector-based healthcare providers were recruited in the study. This has now been clarified in the 'Study Population' section now.<br><br>*'Participants were NHS doctors practising in publicly funded facilities at different stages of training, ranging from trainees to consultants across both community and hospital-based settings.' (Pg 4, lines 124-128)*<br><br>However, we agree that the interoperability challenges would certainly differ between public/private providers, and we have now acknowledged this in the 'Future Research' section as a point for further exploration in follow-on studies.<br><br>*'Another under-explored aspect worth investigating in follow-up studies is interoperability challenges encountered by public and private NHS healthcare providers. Research examining EHR user experience, technical aspects of interoperability, or tangential areas such as*

	implementation sciences and economic analyses can advance the understanding of the value posed by interoperable EHRs [51].' (Pg 15, lines 430-434)
6. Authors could provide examples what they mean by "safety risk for patients" and "posed a potential risk to patient safety"— for clarity and consistency.	Thank you for this comment.<br><br>We agree that providing examples would better highlight what type of safety risks to patients, researchers and doctors are referring to in this study. Text has been amended to both the 'Results' and 'Discussion 'section to better illustrate what is meant by these safety risks.<br><br>*'Similarly, 81.5% (n=335/411) of respondents reported that they felt poor EHR interoperability posed a potential risk to patient safety (e.g., incomplete medical histories, inaccurate medication lists, missing drug allergies), with 19.2% (n=79) describing it as a risk most of the time or always.' (Pg 8, lines 221-224)*<br><br>*'Regarding the perceived impact on patient safety overall, most doctors reported that the limited EHR interoperability they routinely experienced does pose some form of risk (e.g., incomplete medication/allergy lists, missing clinical letters / documentation).' (Pg 12, lines 312-315)*
7. Suggestion for further research: A qualitative component such as semi-structured interviews could enhance understanding of the more nuanced and critical interoperability issues that physicians face. Such methods would allow deeper exploration of the experiences and specific challenges encountered in clinical practice.	Thank you for this comment.<br><br>We certainly agree that employing qualitative methods would be valuable in exploring critical interoperability issues physicians and other healthcare staff encounter, in a more robust manner. We have now added text in the 'Implications to Future Research' section to address this:<br><br>*'Given that other NHS healthcare professionals may be similarly affected by limited EHR interoperability, there is a clear imperative to explore these perspectives, particularly through the employment of qualitative methodologies such as semi-*

	structured interviews. When conducted alongside quantitative methodologies, this would allow for a deeper and more comprehensive interrogation of the experiences and specific challenges encountered in clinical practice.' (Pg 15, lines 425-430)
